# Cross-sectional association between social and demographic factors and disease activity in rheumatoid arthritis

**DOI:** 10.1186/s41927-023-00371-6

**Published:** 2024-01-19

**Authors:** Lei Zhu, Larry W. Moreland, Dana Ascherman

**Affiliations:** 1 Division of Rheumatology and Clinical Immunology, School of Medicine, University of Pittsburgh, BST S723, 200 Lothrop Street, Pittsburgh, PA 15261 USA; 2 Microbial Genomic Epidemiology Laboratory, Center for Genomic Epidemiology, University of Pittsburgh, Pittsburgh, PA USA; 3 Division of Infectious Diseases, University of Pittsburgh School of Medicine, Pittsburgh, PA USA; 4 Division of Rheumatology, School of Medicine, and Orthopedics, University of Colorado, Aurora, CO USA

**Keywords:** Rheumatoid arthritis (RA), Disease activity, Disease activity-C reactive protein (DAS28-CRP), Health assessment questionnaire (HAQ), Social context

## Abstract

**Background:**

This study aimed to assess the association between social factors, demographic parameters, and disease activity among rheumatoid arthritis (RA) patients.

**Methods:**

The University of Pittsburgh Rheumatoid Arthritis Comparative Effectiveness Research (RACER) registry was used for this study and included patients meeting 1987 ACR criteria for RA enrolled between 2010–2015. The registry collected clinical and laboratory data at each visit, permitting the calculation of disease activity measures that included Disease Activity 28-C Reactive Protein (DAS28-CRP). The current study was conducted as a cross-sectional study in which baseline data were used to construct multiple logistic regression models assessing the relationship between disease activity measures (DAS28-CRP), functional capacity (health assessment questionnaire (HAQ)), selected demographic and social factors (occupation, education, income, marital status, race, gender, age, and BMI), and clinical/laboratory variables.

**Results:**

The analyses included 729 patients with baseline DAS28-CRP and social/demographic data. The mean age at enrollment was 59.5 (Standard Deviation (SD) = 12.7) years, 78% were female, and the median RA disease duration was 9.8 (Interquartile Range (IQR): 3.7, 19.1) years. We dichotomized the DAS28-CRP score and defined scores above or below 3.1 as high versus low RA disease activity. Most patients with high RA disease activity (*N* = 326, 45%) had less than a college degree (70%), were not working/retired/disabled (71%), and had an annual income under $50 K (55%). We found that higher body mass index (BMI) (Odds Ratio (OR) = 1.04, 95% CI: 1.01—1.08), longer disease duration (> 2 and < 10 years versus ≤ 2 years of disease) (OR = 0.45, 95% CI: 0.25—0.78), and being retired (OR = 1.74, 95% CI: 1.02—2.98) were associated with RA disease activity.

**Conclusion:**

Increased RA activity may be associated with various social factors, potentially leading to more severe and debilitating disease outcomes. These findings provide evidence to support efforts to monitor disparities and achieve health equity in RA.

## Introduction

Rheumatoid arthritis (RA) is a complex inflammatory autoimmune disease that affects approximately 1% of adult Americans [1]. Epidemiological studies indicate that 71/10,000 people are diagnosed with RA yearly in the US, with significant reductions in quality of life, progressive disability, increased death rate, and increased socioeconomic cost [2, 3]. While previous studies have highlighted considerable heterogeneity in disease progression and disease outcomes, the underlying mechanisms for variations in health risks/outcomes remain unclear [4, 5].

There is mounting evidence that social disparities are associated with RA. According to various surveys (e.g., National Health and Nutrition Examination Survey-III) and other studies conducted in the US and Europe, there is a higher incidence of RA observed for those with lower education levels and socioeconomic status (SES) [6–9]. Additionally, researchers have found that SES and health disparities impact disease activity as well as the clinical course of RA [8–12]. To date, most of the studies focusing on social determinants of health among patients with RA used patient- or physician-reported outcomes (including DAS28) for disease activity or functional status evaluation [8–11, 13, 14]. The validated disease activity index, DAS28-CRP, is one of the most widely used instruments in both clinical trials and daily practice [15]. Accurate use of such validated indices should provide additional insight regarding likely associations between health disparity and disease outcome, particularly for those receiving at least some rheumatology care. In turn, through improved recognition of the potential impact of SES and health disparity on disease outcome, physicians should be able to make better clinical decisions and develop more effective disease management plans.

Consisting of 1,094 prospectively enrolled patients meeting 1987 ACR criteria for RA, the Rheumatoid Arthritis Comparative Effectiveness Research (RACER) cohort offers a unique opportunity to study the impact of social factors on disease activity in greater depth. Of note, this registry includes patient reported outcomes as well as outcome measures assessed by rheumatologists, ensuring higher fidelity determination of RA disease activity, and facilitating more meaningful correlations with social and demographic factors. We therefore sought to determine whether particular social and/or demographic variables were associated with disease activity in order to shed light on the relationship between these factors and risk of poor disease outcome.

## Methods

### Study population

The University of Pittsburgh RACER registry consists of prospectively enrolled RA patients recruited through the University of Pittsburgh Medical Center (UPMC). Since its inception in 2010, RACER has enrolled more than 1,000 patients older than 18 years of age who meet the 1987 ACR diagnostic criteria for RA. The RACER registry protocol was approved by the University of Pittsburgh Institutional Review Board, and all RACER subjects provided informed consent prior to enrollment.

### Assessment of variables

At baseline, we measured RA disease activity by using the physician-derived Disease Activity-C Reactive Protein (DAS28-CRP) scoring system, which includes counting of 28 swollen and tender joints, assessment of global disease activity reported by patients, and measurement of inflammatory markers (i.e., CRP) in the blood; DAS28-CRP = 0.56 x √ [total tender joints]) + (0.28 x √ [total swollen joints]) + (0.36 x (log ([CRP mg/L] + 1))) + (0.014 x ([patient global health × 10])) + 0.96. To define high versus low disease activity, we dichotomized the DAS28-CRP score. While DAS28-CRP scores above 3.1 were considered high disease activity, DAS28-CRP scores less than or equal to 3.1 were interpreted as low disease activity. [16] Health Assessment Questionnaire (HAQ) scores were also assessed as a measure of functional status in our patient cohort [17]. These scores were dichotomized into equal to or greater than mean and less than mean to produce odds ratios for high versus low functional status/quality of life (HAQ ≥ 1.9 vs. < 1.9).

Social determinants assessed in this study included educational level, employment status (working, not working, disabled, or retired), household annual income (stratified by $25,000 increments), and marital status. Demographic and racial factors encompassed age, gender, race and ethnicity. At the baseline visit, social and demographic factors were collected for each patient. Regression models incorporating variables such as sociodemographic factors, clinical data (systolic blood pressure, diastolic blood pressure), biomarker data (i.e., CRP, interleukin 6 (IL-6), rheumatoid factor (RF)), and disease duration were used to assess the association between social/demographic factors and RA disease activity (after adjusting for potential confounding variables, as detailed below).

### Data reduction

We checked missing data patterns for the original 1094 patients in the RACER registry and examined the distribution of demographic characteristics (e.g., sociodemographic and clinical profile variables) between the missing data groups and non-missing data groups. According to our findings, the demographic/clinical characteristics did not differ between the two groups, indicating that the missing data were randomly distributed.

**Fig. 1:**
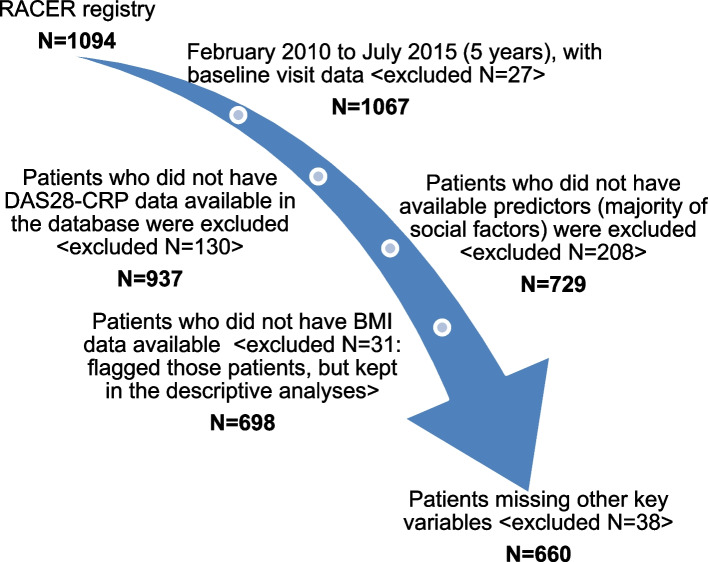
Data reduction process

Based on the availability of baseline DAS28-CRP scores as well as data encompassing demographic and social factors, 729 of the 1094 original RACER patients enrolled between February 2010 and July 2015 were included in our analysis. Because BMI data were missing for more than 300 patients, we obtained BMI data from UPMC electronic medical records (EMR). With those EMR data, 31 patients still had missing BMI. There were 109 patients missing other social/demographic variables (e.g., education level, marital status). Since these missing data related to different variables across patients, these variables were still included in descriptive analyses (Fig. 1).

### Statistical analysis

Statistical tests for parametric and nonparametric variables were conducted. More specifically, Pearson correlation tests for parametric continuous variables and Spearman correlation tests for non-parametric continuous variables were performed. Missing data patterns were examined, and the demographic characteristics as well as data distribution between patients missing and not missing data were compared to confirm that these data were missing at random. All covariates were evaluated for normality. For continuous variables with a normal distribution, means and standard deviations were reported, while medians and interquartile ranges were reported for continuous variables without a normal distribution. When comparing continuous variables, t-tests were used for normally distributed data, while the Wilcoxon test was used for non-parametric data to assess statistical significance. Statistical comparisons of dichotomous variables (high versus low disease activity) were made using Chi-square or Fisher exact tests (if sample size was small).

The association between social and demographic factors and RA disease activity was assessed using bivariate logistic regression models, which included the RA disease activity indicator and selected covariates. Statistical significance was determined by regression coefficients and corresponding *p* values (considered significant when < 0.05), and the associations between social/demographic factors and RA disease activity were presented as odds ratios (OR) with corresponding 95% confidence intervals (95% CI). Covariates included in the final multiple logistic regression models were selected based on 1) univariate and bivariate regression models (*p* < 0.05); 2) backwards selection of covariates; and 3) clinical relevance. In short, we conducted model selection by using a combination of backwards elimination, and we subsequently evaluated whether including those variables into the model had clinical significance. Backwards elimination was performed by comparing the *p*-value with a predetermined significance level of 0.2. If non-significant, the variable was dropped from the model. Using Akaike information criterion (AIC) and likelihood ratio tests, we then determined the best fitting model. As a complement to these analyses, we also constructed models using HAQ as the dependent variable and then evaluated the association between social/demographic factors and HAQ scores (high vs. low). Statistical significance was determined by a 2-sided *p*-value < 0.05. Statistical computing was conducted using SAS version 9.4 (SAS Institute Inc., Cary, NC, USA).

## Results

Study participants included 729 adults with RA DAS28-CRP measurements as well as recorded baseline demographic and social factors needed to evaluate associations between these variables and RA disease activity. The mean age in this population was 59.5 ± 12.7 years, and 78% were female—with a median RA disease duration of 9.8 (IQR: 3.7, 19.1) years (Table 1). While the mean baseline DAS28-CRP score was 3.1 (SD = 1.36, range: 1–7.9), the mean baseline HAQ score was 1.9 (SD = 1.70, range: 0–8.7). Approximately 45% of patients in our final study cohort had evidence of high RA disease activity (DAS28-CRP > 3.1) (*N* = 326), paralleling the number/percentage of patients (322 patients, 44%) with high HAQ scores (HAQ > 1.9). Overall, the correlation between DAS28-CRP and HAQ scores was modest (*r* = 0.56, *p* < 0.05).

**Table 1:** Baseline demographic and social characteristics of RACER cohort segregated by RA disease activity

**Category**	**Level**	**Total n, %**	**High DA**^a^** n, %**	**Low DA n, %**	**Chi-square *p*-value**
**All**		729, 100%	326, 45%	403, 55%	
**Gender**	Female	569, 78%	261, 80%	308, 76%	0.2385
**Age at Baseline Visit**	N, Mean (sd)	729, 59.5 (12.7)	326, 59.7 (12.8)	403, 59.3 (12.7)	0.6353
**BMI (kg/m**^**2**^**)**	N, Mean (sd)	698, 29.2 (6.3)	316, 30.1 (6.8)	382, 28.5 (5.7)	0.0011*
**Race**	White	650, 89%	281, 86%	369, 92%	0.0598
**Smoking**	Yes	103, 14%	56, 17%	47, 12%	0.0347
**Drinking**	Yes	379, 52%	152, 47%	227, 56%	0.0091*
**Education Level**	< High School	33, 5%	21, 6%	12, 3%	
	High School	433, 59%	208, 64%	225, 56%	
	College	164, 22%	58, 18%	106, 26%	
	Graduate	98, 13%	39, 12%	59, 15%	0.0037*
**Employment Status**	Working	242, 33%	87, 27%	155, 38%	
	Retired	308, 42%	145, 44%	163, 0%	
	Not working	64, 9%	30, 9%	34, 8%	
	Disabled	104, 14%	59, 18%	45, 11%	0.0024*
**Marital Status**	Married	414, 57%	171, 52%	243, 60%	
	Single	101, 14%	46, 14%	55, 14%	
	Separated; Divorced; Widowed	214, 29%	109, 33%	105, 26%	0.0698
**Annual income**	0-25 k	177, 24%	99, 30%	78, 19%	
	25 k-50 k	172, 24%	74, 23%	98, 24%	
	50 k-75 k	98, 13%	45, 14%	53, 13%	
	75 k-100 k	65, 9%	22, 7%	43, 11%	
	> 100 k	62, 9%	20, 6%	42, 10%	0.0028*
**Private Insurance**	Yes	631, 87%	260, 80%	371, 92%	< 0.0001*
**Medicaid**	Yes	69, 9%	45, 14%	24, 6%	0.0003*

Number of missing for the above variables’ bivariate analyses are: Race (missing *N* = 10); Employment (missing *N* = 11); Income (missing *N* = 155); Smoking, and Education (missing *N* = 1, for each)

^a^*DA* Disease activity, *High DS* DAS28-CRP > 3.1, *Low DS* DAS28-CRP <  = 3.1

^*^Significant at *p* < 0.05

Analysis of various clinical and laboratory variables in this cohort demonstrated statistical associations between DAS28-CRP and BMI, smoking, education level, employment status, annual income, possession of private insurance or Medicaid, disease duration, RF, and IL-6. Pearson and Spearman correlation tests demonstrated modest correlations between CRP and DAS28-CRP (*r* = 0.52, *p* < 0.05), suggesting that these variables could not be considered independent in subsequent construction of logistic regression models.

More specifically, among individuals who met the DAS28-CRP threshold for high disease activity (DAS28-CRP > 3.1), 70% had less than a college degree, 53% had an annual income < $50 K, and 71% were either not working, retired, or disabled. Patients with higher DAS28-CRP scores were more likely to be African American, unemployed (retired, not working, or disabled) with low annual income (< $50 K) and receiving Medicaid. Additional clinical and laboratory features of the high DAS28-CRP subgroup included greater BMI, reduced frequency of current drinking, elevated CRP levels, higher RF, higher IL-6 levels, and disease duration longer than 10 years. There were no differences in age, gender, marital status, or medication use between high and low RA disease activity subgroups (Table 1 and Table 2).

**Table 2:** Baseline clinical characteristics of RACER cohort segregated by RA disease activity

**All**	**Total n, %**	**High DA**^a^**, n, %**	**Low DA n, %**	**Chi-square *p*-value**
	**729, 100%**	**326, 45%**	**403, 55%**	
**CRP: Abnormal**	241, 33%	170, 52%	71, 18%	< 0.0001*
**Disease duration**				
**0–2 years**	114, 16%	61, 19%	53, 13%	
** > 2 and < 10 years**	253, 35%	98, 30%	155, 38%	
** > 10 years**	360, 49%	167, 51%	193, 48%	0.0221*
**Medication current use**				
**Methotrexate**	720, 98.8%	323, 99%	397, 99%	0.449
**Leflunomide**	72, 10%	35, 11%	37, 9%	NA
**Biologic agent**	275, 38%	95, 29%	180, 45%	1
**Steroid**	720, 98.8%	323, 99%	397, 99%	0.449
	**n, median (IQR)**			**Wilcoxon *p*-value**
**RF**	683, 44 (20, 191)	305, 63 (21, 345)	378, 36 (20, 136)	0.0002*
**Anti-CCP**^b^	728, 41 (2, 206.5)	326, 45.3 (2, 224)	402, 35 (2, 196)	0.3972
**IL-6**	694, 2 (2, 9)	311, 5 (2, 12.3)	383, 2 (2, 6.4)	< .0001*

Number of missing for the above variables’ bivariate analyses are disease duration (missing *N* = 2); Methotrexate (missing *N* = 9); Leflunomide (missing *N* = 657); Biologic agent (missing *N* = 454); Steroid (missing *N* = 9)

^a^DA: disease activity. High DS: DAS28-CRP > 3.1; Low DS: DAS28-CRP <  = 3

^b^Anti-CCP, Anti-Cyclic Citrullinated Peptide antibodies

^*^Significant at *p* < 0.05

We next developed multiple logistic regression models to further examine the relationship between disease activity and selected clinical, demographic, and laboratory variables (Table 3). Model selections were based on statistical tests (e.g., *p* values, AIC) as well as clinical considerations (i.e., whether included variables made sense clinically). Unadjusted models for each covariate were built, demonstrating that smoking, abstinence from alcohol, lower education level, annual income less than $25,000, disease duration < 2 years, and higher BMI were significantly associated with high RA disease activity (as measured by DAS28-CRP). In unadjusted models, marital status, age, gender, and race showed no associations with higher RA disease activity.

**Table 3:** Regression models assessing variables related to RA disease activity

	**Unadjusted Model**		**Adjusted Model**^a^	
	**OR**	**95% CI**	**OR**	**95% CI**
**Female vs. Male**	1.24	0.87—1.77	NA	NA
**Age**	1.00	0.99—1.01	0.99	0.97—1.00
**Black vs. White**	1.61	0.98—2.65	NA	NA
**Smoking**	1.57	1.03—2.38	1.42	0.85—2.35
**Drinking**	0.68	0.51—0.91	0.77	0.53—1.12
**Education**			NA	NA
** < High School**	Reference			
**High School**	0.53	0.25—1.10		
**College**	0.31	0.14—0.68		
**Graduate**	0.38	0.17—0.86		
**Employment**				
**Working**	Reference		Reference	
**Retired**	1.59	1.12—2.24	1.74	1.02—2.98
**Not working**	1.57	0.90—2.74	1.09	0.53—2.23
**Disabled**	2.34	1.46—3.73	1.78	0.98—3.24
**Marital Status**			NA	NA
**Married**	Reference			
**Single**	1.19	0.77—1.84		
**Separated; Divorced; Widowed**	1.48	1.06—2.06		
**Annual income**				
**0-25 K**	Reference		Reference	
**25-50 K**	0.60	0.39—0.91	0.71	0.45—1.14
**50-75 K**	0.67	0.41—1.10	0.97	0.55—1.71
**75-100 K**	0.40	0.22—0.73	0.56	0.29—1.12
** > 100 K**	0.38	0.20—0.69	0.49	0.24—1.12
**BMI**	1.04	1.02—1.07	1.04	1.01—1.08
**Disease duration**				
**0–2 years**	Reference		Reference	
** > 2 and < 10 years**	0.55	0.35—0.86	0.45	0.25—0.78
** > 10 years**	0.75	0.49—1.15	0.77	0.45—1.32

^a^Model adjusted for age, BMI, smoking, drinking, employment status, annual income, and disease duration

After adjusting for those variables that were clinically relevant and statistically associated with outcome, retired working status (OR = 1.74; 95% CI: 1.02—2.98), and greater BMI (OR = 1.04; 95% CI: 1.01—1.08) remained significantly associated with higher RA disease activity. Those patients with longer disease duration (2–10 years compared to < 2 years) at baseline were less likely to have high disease activity (OR = 0.45; 95% CI: 0.25—0.78) (Table 3). The variable inflation factors for both the unadjusted and selected adjusted models were low (< 2), and the residuals were randomly distributed. Model diagnostic tests were conducted, and the results suggested that the models fit well (data not shown).

In our supporting analyses adjusted for selected covariates and using HAQ score as the outcome index, retired working status (OR = 2.02; 95% CI: 1.14—3.60), disabled status (OR = 9.14; 95% CI: 4.37—19.10), lower annual household income, and greater BMI (OR = 1.05; 95% CI: 1.02—1.09) remained significantly associated with greater HAQ scores (Table 4)—paralleling observations with DAS28-CRP/disease activity as the outcome measure (Table 4).

**Table 4:** Regression models assessing variables impacting RA HAQ

	**Unadjusted Model**		**Adjusted Model**^a^	
	**OR**	**95% CI**	**OR**	**95% CI**
**Female vs. Male**	1.69	1.17—2.44	1.57	0.96—2.59
**Age**	1.01	0.99—1.02	0.99	0.97—1.01
**Black vs. White**	1.44	0.87—2.41	1.19	0.67—2.12
**Smoking**	1.74	1.14—2.66	0.71	0.47—1.07
**Drinking**	0.45	0.33—0.60	0.77	0.53—1.12
**Education**			NA	NA
** < High School**	Reference			
**High School**	0.49	0.23—1.04		
**College**	0.31	0.14—0.68		
**Graduate**	0.22	0.09—0.50		
**Employment**				
**Working**	Reference		Reference	
**Retired**	2.31	1.61—3.32	2.02	1.14—3.60
**Not working**	1.34	0.74—2.41	0.67	0.30—1.49
**Disabled**	12.19	6.82—21.81	9.14	4.37—19.10
**Marital Status**			NA	NA
**Married**	Reference			
**Single**	2.25	1.44—3.50		
**Separated; Divorced; Widowed**	2.05	1.46—2.88		
**Annual income**				
**0-25 K**	Reference		Reference	
**25-50 K**	0.39	0.25—0.60	0.52	0.31—0.86
**50-75 K**	0.31	0.18—0.51	0.59	0.32—1.08
**75-100 K**	0.16	0.08—0.31	0.25	0.12—0.53
** > 100 K**	0.13	0.07—0.27	0.24	0.10—0.54
**BMI**	1.05	1.03—1.08	1.05	1.02—1.09
**Disease duration**				
**0–2 years**	Reference		Reference	
** > 2–10 years**	2.45	1.44—3.50	0.65	0.35—1.21
** > 10 years**	2.05	1.46—2.88	0.91	0.50—1.65

^a^Model adjusted for age, gender, BMI, smoking, drinking, employment status, annual income, and disease duration

## Discussion

In this study, we evaluated the associations between social and demographic factors and disease activity among a large Pittsburgh-based cohort of patients with rheumatoid arthritis. Our study included a group of RA patients with comprehensive health outcome assessments as well as well-annotated demographic and social characteristics, allowing us to investigate associations and fill gaps not addressed by previous studies. Overall, our analyses indicated that among RA patients in this Pittsburgh RACER cohort, smoking, abstinence from alcohol, lower education level, being retired or disabled, being separated/divorced/widowed, having an annual income under $25 K, higher BMI, and shorter RA disease duration (≤ 2 vs. 2–10 years) were independently associated with high RA disease activity (as defined by DAS28-CRP scores). Following adjustment for confounders, retired work status, higher BMI, and shorter RA disease duration remained significantly associated with high RA disease activity.

Patients with rheumatoid arthritis suffer from a large disease burden [18]. While more new treatments (e.g., disease-modifying antirheumatic drugs) have become available to RA patients and have led to dramatic improvements in health outcomes, existing health disparities result in uneven distribution of these gains across the population. However, better understanding of the disparity issues in patients with rheumatoid arthritis should facilitate interventions for those vulnerable groups.

Previous investigations evaluating the impact of demographic and social factors on RA disease progress have been limited [8, 13]. For example, Greenberg et al. reported that disparities correlate with disease activity and clinical outcomes of RA patients cross-sectionally and longitudinally, but this study did not present data examining the association between other social factors (such as income level/socioeconomic status) and RA outcomes [8]. While another study by Izadi et. al. evaluating social factors as determinants of disease outcome suggested that socioeconomic disparities were associated with declines in functional status among patients with RA in cross-sectional and longitudinal analyses [13], this study did not further investigate the association between social and/or demographic factors and RA disease activity. Our study was able to include a broader range of social and demographic factors than previous studies to evaluate the association between those explanatory variables and RA disease activity, making quantitative evaluation more reliable.

Additionally, HAQ scores were obtained to further evaluate the association between social/ demographic factors and functional status as another patient-reported outcome (Table 4)–permitting comparison to social/demographic variables associated with disease activity. In fact, the observed associations with HAQ scores mirrored the demonstrated relationship between social factors and high RA disease activity as measured by DAS28-CRP, supporting our conclusions.

Consistent with our findings from the UPMC RACER cohort, a relationship between social and demographic parameters and RA disease activity has been reported in a few other studies [8, 10, 11]. Our findings reinforce previous studies demonstrating that social factors are associated with rheumatoid arthritis disease activity. Unlike these previous studies, however, our study did not demonstrate that race, marital status, and education level were linked to higher disease activity, after adjusting for confounders. This discrepancy likely indicates that single SES factors do not completely account for health disparities, unless combined with other factors such as income and employment indicators. Another possible reason for those variables falling out in our multiple regression models could be the small sample size of certain subgroups. As an example, the RACER cohort includes a relatively small number of African Americans (reflecting the overall demographic profile of Pittsburgh), with only 14% in the high disease activity group and 8% in the low disease activity group. This relatively limited racial variability may therefore explain the lack of association between race and disease activity in our cohort.

In our study, marital status (separated, divorced, or widowed vs. married) at baseline visit was associated with higher RA disease activity; however, the association was not significant when comparing those who previously married to those who never married/remained single. Furthermore, after further adjusting for age, none of these potential associations remained significant (ORs less than 1)–indicating that the apparent association between RA disease activity and marital status at baseline likely reflects uncontrolled confounding by age, rather than influence of social burden (data not shown). We therefore did not include marital status into our final model.

Among the most interesting findings of our study, longer RA disease duration (> 2 and < 10 years) was associated with lower disease activity (relative to ≤ 2 years’ disease duration). One possible explanation for this finding could be that patients with 2–10 years’ disease duration at baseline had received a longer duration of treatment and therefore had more stable disease compared to those who were more recently diagnosed with RA. Interestingly, disease duration > 10 years was not associated with lower disease activity (compared to ≤ 2 years’ disease), possibly because the older age of patients in this subcategory counteracted factors associated with lower disease activity in individuals with an intermediate disease duration of 2–10 years.

The main strengths of this study included the availability of many rheumatoid disease clinical characteristics, SES indicators, and relevant covariates in the clinical registry and EMR. This degree of annotation allowed us to conduct thorough analyses supported by the large sample size of our RA cohort and appropriate adjustment for key confounder variables. Additionally, the clinical measurements in this study were made by rheumatologists, providing better RA diagnostic and clinical assessments compared to data derived from administrative databases. To the best of our knowledge, our study is one of the largest US studies to evaluate the association between RA disease activity and a wide range of social and demographic factors measured at an individual level by rheumatologists.

Despite its size, this single center study was characterized by relatively limited social/racial variability among cohort constituents, potentially limiting the generalizability of our findings. Another major limitation of this study was that we only looked at baseline data for these analyses. As RA disease activity changes over time, further study with time-varying RA disease activity measures as outcomes will be needed to validate our findings. In addition, cross-sectional studies cannot support causality for observed associations, particularly because reverse causality may exist. Because longitudinal observational studies incorporating both RA disease activity indices and social factors measured over time can potentially counteract many of these limitations, future goals include examination of clinical visit data among those patients with RA over the 5-year follow-up period in the RACER registry to further evaluate the relationship between disease activity and demographic/social factors. Ultimately, these analyses will be crucial to improve treatment efficacy and personalize patient management.

## Conclusions

Overall, our findings indicate that significant differences in RA disease activity exist among different social groups (e.g., with different employment status). Although causality cannot be ascertained from this study, social disparity could lead to delays in seeking treatment and lack of early care among patients with rheumatoid arthritis, ultimately resulting in more serious and debilitating disease outcomes.

## Data Availability

The datasets used and/or analyzed during the current study available from the corresponding author on reasonable request.

## Abbreviations

RA: Rheumatoid arthritis
RACER: Rheumatoid Arthritis Comparative Effectiveness Research
DAS28-CRP: Disease Activity 28-C Reactive Protein
SD: Standard Deviation
IQR: Interquartile Range
BMI: Body Mass Index
HAQ: Health Assessment Questionnaire
SES: Socioeconomic Status
UPMC: University of Pittsburgh Medical Center
IL-6: Interleukin 6
RF: Rheumatoid Factor
Anti-CCP: Anti-Cyclic Citrullinated Peptide Antibodies
EMR: Electronic Medical Records
OR: Odds Ratios
95% CI: 95% Confidence Interval
AIC: Akaike Information Criterion
DA: Disease Activity
NA: Not Available
